# Circulating exosomal tsRNAs: Potential biomarkers for large artery atherosclerotic stroke superior to plasma tsRNAs

**DOI:** 10.1002/ctm2.1194

**Published:** 2023-01-31

**Authors:** Kaiying Yang, Qi Xiao, Kun Wang, Jie Zhao, Rongyao Hou, Xudong Pan, Xiaoyan Zhu

**Affiliations:** ^1^ Department of Neurology The Affiliated Hospital of Qingdao University Qingdao China; ^2^ Department of Neurology The Affiliated Hiser Hospital of Qingdao University Qingdao China; ^3^ Department of Critical Care Medicine The Affiliated Hospital of Qingdao University Qingdao China

Dear editor,

Ischemic stroke (IS) occurrence increased the most (226.5%) among all types of strokes from 1990 to 2019.^1^ Meanwhile, large‐artery atherosclerotic (LAA) stroke forms the most significant proportion of IS.^2^ Advanced neuroimaging technologies have been widely applied to diagnose stroke; however, MRIs, which are more accurate, are only readily available in some centres. Exosomal tsRNAs, with their more stable properties and traceability, show great potential as biomarkers for disease diagnosis.^3^, ^4^ Hence, by comparing exosomal tsRNA levels in different types of IS, we indicated that targeting circulating exosomal tsRNAs is a potential strategy for LAA stroke diagnosis, short‐term prognosis assessment and plaque stability detection, superior to detecting plasma tsRNAs.

A total of 506 peripheral blood samples (LAA: *n* = 153; SAO: *n* = 110; AS: *n* = 105; NC: *n* = 138) were collected at the Affiliated Hospital of Qingdao University in strict accordance with the inclusion and exclusion criteria (Tables S1 and S2). As shown in the working flow chart in Figure 1A,B, all subjects were divided into three cohorts, among which three LAA patients and three healthy controls were randomly selected. The remaining subjects were divided into a validation set (30 LAA: 30 NC) and a replication set (120 LAA: 105 NC: 110 SAO). Exosomes were extracted and validated according to the protocol of the International Society for Extracellular Vesicles.^5^ Figures 1C‐E show that exosomes are bilayer vesicles of 40–110 nm in diameter with protein markers CD9, CD63 and TSG101 on their surface. RNA‐seq results reported 15 significantly different exosomal tsRNAs in the control and LAA groups, of which three were up‐regulated, and 12 were down‐regulated in the LAA group (Figure 1F). GO and KEGG analysis showed that the downstream pathways are mainly focused on metabolism, inflammation and immunity, which are related to the pathological process of AS (Figure S2).

**FIGURE 1:**
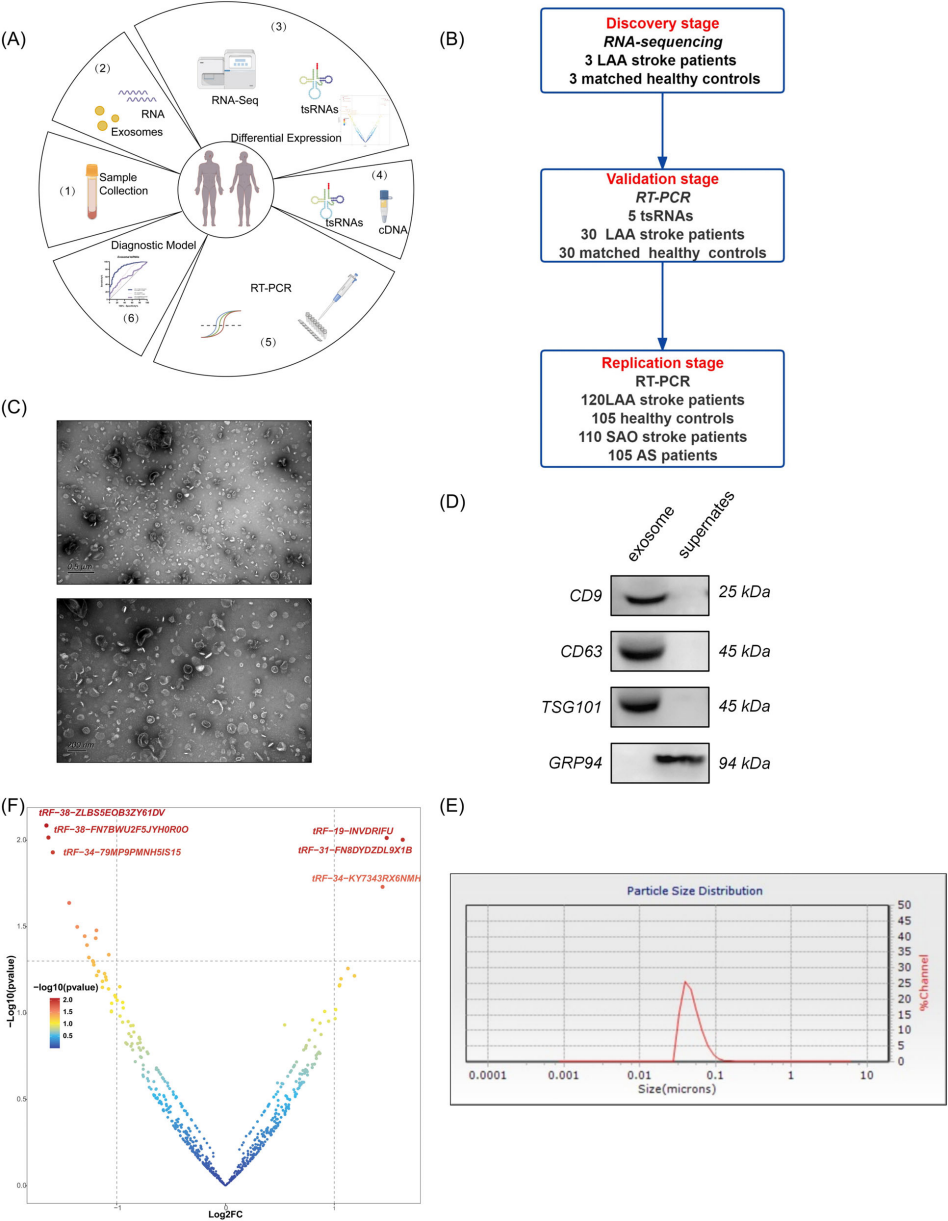
Workflow, characteristics of plasma‐derived exosomes from participants and expression profiles of circulating exo‐tsRNAs. (A) Workflow of the study, including clinical sample collection and processing, discovery stage of tsRNAs sequencing, detection and diagnosis. (B) Workflow of the discovery, validation and replication stage of circulating exosomal tsRNAs. (C) Electron micrograph of the exosomes. (D) Western blot showing that CD9, CD63 and TSG101 were detected in the enriched exosome samples isolated from plasma, while the negative exosomal marker GRP94 was not detected in these samples. (E) NTA of the exosomes size distribution. (F) Volcano plots of differential exosomal tsRNAs in LAA stroke. The figure shows 15 exosomal tsRNAs with the most significant differential expression.

Combined with the foldchange and functional enrichment results, we finally selected five tsRNAs for subsequent verification: up: tRF‐19‐INVDRIFU, tRF‐31‐FN8DYDZDL9X1B; down: tRF‐34‐79MP9PMNH5IS15, tRF‐36‐FN7BWU2F5JYH0RE, tRF‐38‐Q99P9P9NH57S36D1 (Primer sequences: Table S2). We detected the expression of the tsRNAs mentioned above by RT‐qPCR in the validation set (Figure 2A). The between‐group difference results initially confirmed that exosomal tsRNAs are expected to diagnose LAA stroke. Based on this, we further performed a large sample replicate validation. At this stage, we also included the SAO group to exclude the possibility of changes in tsRNAs expression due to acute ischemic stroke. As shown in Figure 2B, except for tRF‐36‐FN7BWU2F5JYH0RE, the other tsRNAs met the conditions of statistically significant difference between the LAA and NC groups and between the LAA and SAO groups. Univariate and multivariate regression analyses (Tables S4 and S5; Figure 2C) incorporating known risk factors (Low‐density lipoprotein, hypertension, diabetes and smoking) showed that tRF‐19‐INVDRIFU was consistently a vital independent risk factor for LAA stroke (tRF‐19‐INVDRIFU: OR = 1.14, 95% CI: 1.058‐1.228, *p* = 0.001; tRF‐38‐Q99P9P9NH57S36D1: OR = 0.529, 95% CI: 0.383‐0.731, *p* < 0.001). Further receiver operating characteristic curve analysis showed an AUC of 0.8469 for the combined diagnostic model, which was superior to the single index tRF‐19‐INVDRIFU (NRI = 0.196) (Figure 2D).

**FIGURE 2:**
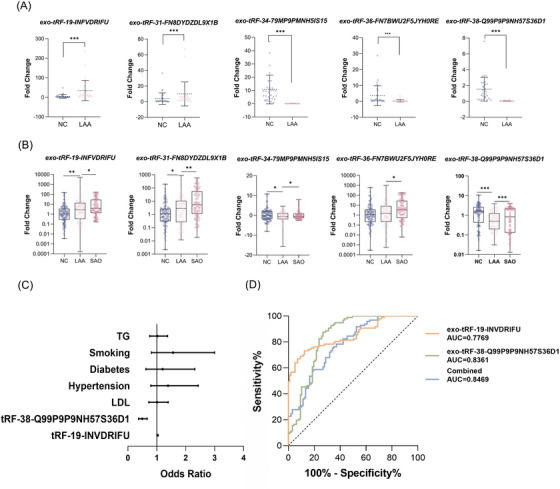
Expression of exosomal tsRNAs as novel biomarkers for LAA stroke. (A) Validation of exo‐tRF‐19‐INVDRIFU, exo‐tRF‐31‐FN8DYDZDL9X1B, exo‐tRF‐34‐79MP9PMNH5IS15, exo‐tRF‐36‐FN7BWU2F5JYH0RE and exo‐tRF‐38‐Q99P9P9NH57S36D1 in NC and LAA groups. (B) Replication of exo‐tRF‐19‐INVDRIFU, exo‐tRF‐31‐FN8DYDZDL91B, exo‐tRF‐34‐79MP9PMNH5IS15, exo‐tRF‐36‐FN7BWU2F5JYH0RE and exo‐tRF‐38‐Q99P9P9NH57S36D1 in NC, LAA and SAO groups. (C) Multivariate logistic regression analysis for exosomal tsRNAs. (D) ROC curves for exo‐tRF‐19‐INVDRIFU, exo‐tRF‐38‐Q99P9P9NH57S36D1 and the combined logistic regression model for both.

Plasma tsRNAs can originate from various cells, but this free form makes tracing its origin difficult. In contrast, the phospholipid bilayers of exosomes have many protein markers that reflect their source cells.^6^ The vesicle structure also isolates tsRNAs from biological enzymes in plasma, making it more stable.^6^ To prove whether plasma tsRNAs could have the same effect as exosomal tsRNAs, RT‐qPCR was used to evaluate the difference in plasma tsRNAs among the LAA, NC and SAO groups. As shown in Figure 3A, only plasma tRF‐31‐FN8DYDZDL91B and plasma tRF‐38‐Q99P9P9NH57S36D1 showed significant differences in expression levels. We further analysed the correlation between plasma and exosomal tsRNAs (Figure 3B) and found that they were not well‐correlated. Moreover, the AUC value of tsRNAs isolated directly from plasma in distinguishing LAA patients from healthy controls was significantly lower than that of exosomal tsRNAs (plasma tRF‐38‐Q99P9P9NH57S36D1: AUC = 0.6; exo‐tRF‐38‐Q99P9P9NH57S36D1: AUC = 0.8361; IDI = 0.743) (Figure 3C).

**FIGURE 3:**
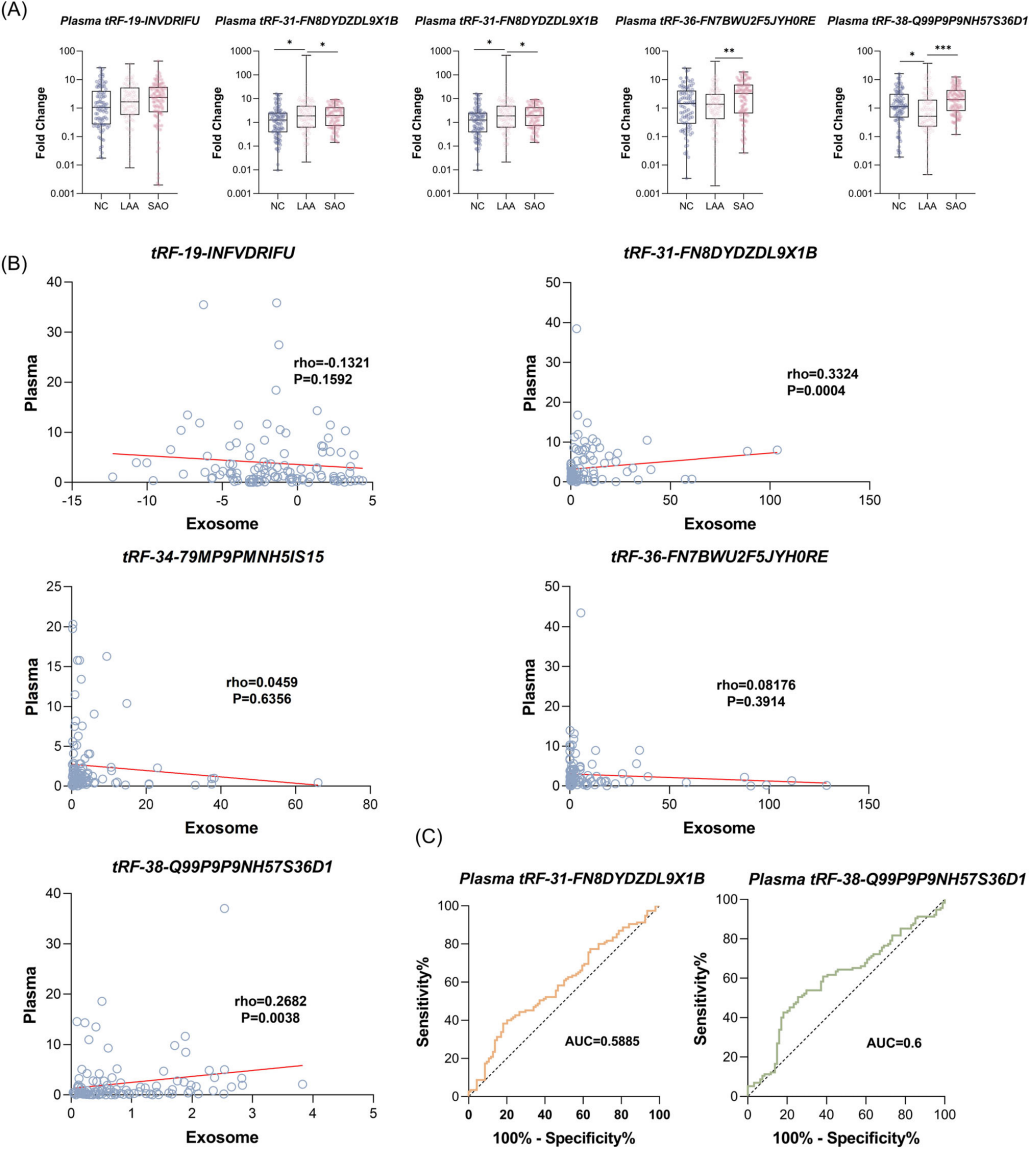
Diagnostic efficacy comparison between exosomal tsRNAs and plasma tsRNAs. (A) Expression of plasma tRF‐19‐INVDRIFU, tRF‐31‐FN8DYDZDL9X1B, tRF‐34‐79MP9PMNH5IS15, tRF‐36‐FN7BWU2F5JYH0RE and tRF‐38‐Q99P9P9NH57S36D1 in NC, LAA and SAO groups. (B) Correlation between exosomal tsRNAs and plasma tsRNAs. (C) ROC curves of the plasma‐tRF‐31‐FN8DYDZDL9X1B and plasma‐tRF‐38‐Q99P9P9NH57S36D1.

Subsequently, we also assessed the correlation between exosomal tsRNAs and LAA stroke severity and quantified it according to NIHSS, with higher scores indicating more severe disease.^7^ We found that the expression level of exo‐tRF‐19‐INVDRIFU was positively correlated with NIHSS, while that of exo‐tRF‐38‐Q99P9P9NH57S36D1 was negatively correlated (Figure 4A). mRS Score is a widely used quantitative standard for prognosis assessment in clinics (favourable: 0‐2; poor: 3‐6).^8^ We analysed the relationship between exosomal tsRNAs and short‐term prognosis using the mRS Score 1 month after discharge as a reference for short‐term prognosis (Figure 4B). The expression level of exo‐tRF‐19‐INVDRIFU (*p* = 0.006) was significantly higher in the poor prognosis group than in the good prognosis group, whereas tRF‐38‐Q99P9P9NH57S36D1 showed the opposite trend.

**FIGURE 4:**
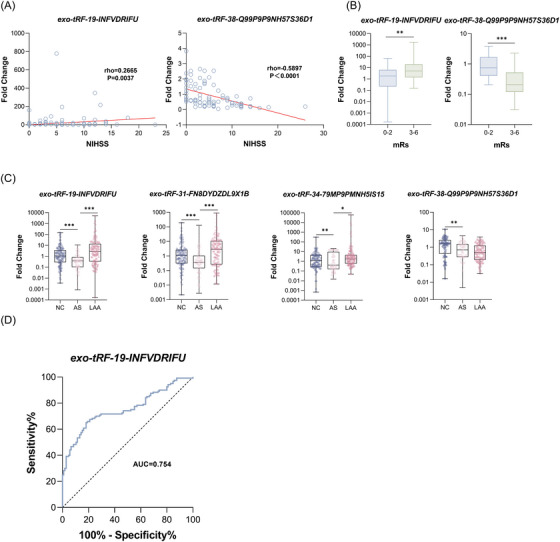
Relationship between exosomal tsRNA and LAA stroke severity, prognosis and plaque stability. (A) Correlation analysis between NIHSS score and exosomal tsRNAs. (B) Differences in exosomal tsRNAs expression between different prognostic groups. (C) Expression of exo‐tRF‐19‐INVDRIFU, exo‐tRF‐31‐FN8DYDZDL9X1B, exo‐tRF‐34‐79MP9PMNH5IS15 and exo‐tRF‐38‐Q99P9P9NH57S36D1 in NC, LAA and AS groups. (D) ROC curve of exosomal tsRNAs for predicting unstable plaques.

The rupture of unstable plaques is the leading cause of LAA stroke.^9^ We included the AS group in duplicate cohorts to determine whether exosomal tsRNAs could be novel biomarkers for predicting unstable plaques (Figure 4C). Interestingly, exo‐tRF‐19‐INVDRIFU was an independent risk factor (OR = 1.436, *p* < 0.001) for plaque rupture. ROC curves are shown in Figure 4D, with an AUC of 0.754. It also performed well in assessing LAA stroke severity and short‐term outcome.

In a nutshell, our study indicated that targeting circulating exosomal tsRNAs is a potential strategy for diagnosing and preventing LAA stroke. Exosomal tsRNAs differed better in differentiating LAA stroke from other groups than plasma tsRNAs. Notably, combined tRF‐19‐INVDRIFU and tRF‐38‐Q99P9P9NH57S36D1 had greater diagnostic efficacy. In addition, exo‐tRF‐19‐INVDRIFU showed an advantage in assessing short‐term poor prognostic grade stroke severity. More to the point, exo‐tRF‐19‐INVDRIFU contributes to assessing plaque rupture risk, which is crucial for early warning of LAA stroke.

## CONFLICT OF INTERESTS

The authors declare no conflict of interests.

## Supporting information

Supporting Information
