# Supplementary material for: Circulating exosomal tsRNAs: Potential biomarkers for large artery atherosclerotic stroke superior to plasma tsRNAs
Source: Clin Transl Med. 2023 Jan 31;13(2):e1194. doi: 10.1002/ctm2.1194 (PMC9889266; doi:10.1002/ctm2.1194)
Supplement: Supplementary file 1 — Supporting Information [file CTM2-13-e1194-s001.docx]

# Supplementary material

**Table S1**. **Baseline of the subjects.** (TG, triglycerides; TC, total cholesterol; LDL, low-density lipoprotein).

| Characteristic | NC  （n=138） | SAO  （n=110） | AS  （n=105） | LAA  （n=153） | P-value  （NCvsLAA） |
| --- | --- | --- | --- | --- | --- |
| Age, mean  (SD), y | 64(10.5) | 63(11.5) | 70(10.6) | 63(9.7) | 0.446 |
| Female, n (%) | 67(48.6%) | 38(34.5%) | 58(55.2%) | 70(45.8%) | 0.633 |
| **Risk factors, n (%)** |  |  |  |  |  |
| Hypertension | 77(55.8%) | 52(55.9%) | 70(66.7%) | 95(62.1%) | 0.345 |
| Smoking history | 103(74.6%) | 40(36.4%) | 19(18.1%) | 55(35.9%) | 0.032* |
| Diabetes mellitus | 29 (21%) | 29(26.4%) | 35(33.3%) | 42(27.5%) | 0.228 |
| TG, mean (SD) | 1.9(1.4) | 1.4(1.0) | 1.5(0.8) | 1.5(1.1) | 0.007** |
| TC, mean (SD) | 4.6(0.9) | 4.4(1.0) | 4.5 (1.3) | 4.5(1.1) | 0.211 |
| LDL, mean (SD) | 2.6(0.7) | 2.7(0.8) | 2.6(0.9) | 2.6(0.9) | 0.856 |

**Table S2. Inclusion and exclusion criteria**

| **Inclusion criteria** | **Exclusion criteria** |
| --- | --- |
| AIS diagnosed by craniocerebral CT or MRI | severe heart disease |
| definite diagnosis within 3 days after the onset of symptoms | other systemic diseases |
|  | tumors |
|  | stroke with other TOAST subtypes |
|  | cerebral embolism due to embolus detachment |

**Table S3. The primer sequences of RT-qPCR**

| **tsRNAs** | **Primer Sequences (5’ → 3’)** |
| --- | --- |
| tRF-19-INVDRIFU | CGGATGTTTAGACGGGCTA |
| tRF-31-FN8DYDZDL9X1B | AGATTTCAACTTAACTTGACCGCTCTGACCA |
| tRF-34-79MP9PMNH5IS15 | TAGTGTAGCGGTTATCACATTCGC |
| tRF-36-FN7BWU2F5JYH0RE | GAATCTGACAACAGAGGCTTACGACC |
| tRF-38-Q99P9P9NH57S36D1 | GTAGTGGTTATCACGTTCGCCTCAC |

**Table S4.** **Binary logistic regression analysis between LAA group and NC group**

| **Univariate logistic regression analysis** | **OR (95%CI)** | **P-value** |
| --- | --- | --- |
| tRF-19-INVDRIFU | 1.142 (1.064-1.226) | ＜0.001** |
| tRF-31-FN8DYDZDL9X1B | 1.010(0.997-1.023) | 0. 151 |
| tRF-34-79MP9PMNH5IS15 | 1.004(0.997-1.010) | 0.270 |
| tRF-38-Q99P9P9NH57S36D1 | 0.473(0.353-0.633) | ＜0.001** |
| **Multivariate logistic regression analysis** |  |  |
| tRF-19-INVDRIFU | 1.14(1.058-1.228) | 0.001 |
| tRF-38-Q99P9P9NH57S36D1 | 0.529(0.383-0.731) | ＜0.001** |
| Gender | 1.587(0.888-2.837) | 0.119 |
| LDL | 1.004(0.725-1.391) | 0.979 |
| Hypertension | 1.387(0.791-2.432) | 0.253 |
| Diabetes | 1.2(0.622-2.313) | 0.586 |
| Smoking | 1.562(0.814-2.996) | 0.18 |

**Table S5. Binary logistic regression analysis between LAA group and AS group**

| **Univariate logistic regression analysis** | **OR (95%CI)** | **P-value** |
| --- | --- | --- |
| tRF-19-INVDRIFU | 1.353(1.179-1.554) | ＜0.001** |
| tRF-31-FN8DYDZDL9X1B | 1.044(1.007-1.083) | 0.021* |
| tRF-34-79MP9PMNH5IS15 | 1.009(0.988-1.030) | 0.4 |
| tRF-38-Q99P9P9NH57S36D1 | 0.745(0.553-1.006) | 0.054 |
| **Multivariate logistic regression analysis** |  |  |
| tRF-19-INVDRIFU | 1.436(1.201-1.718) | ＜0.001** |
| tRF-31-FN8DYDZDL9X1B | 0.969(0.914-1.027) | 0.284 |

**
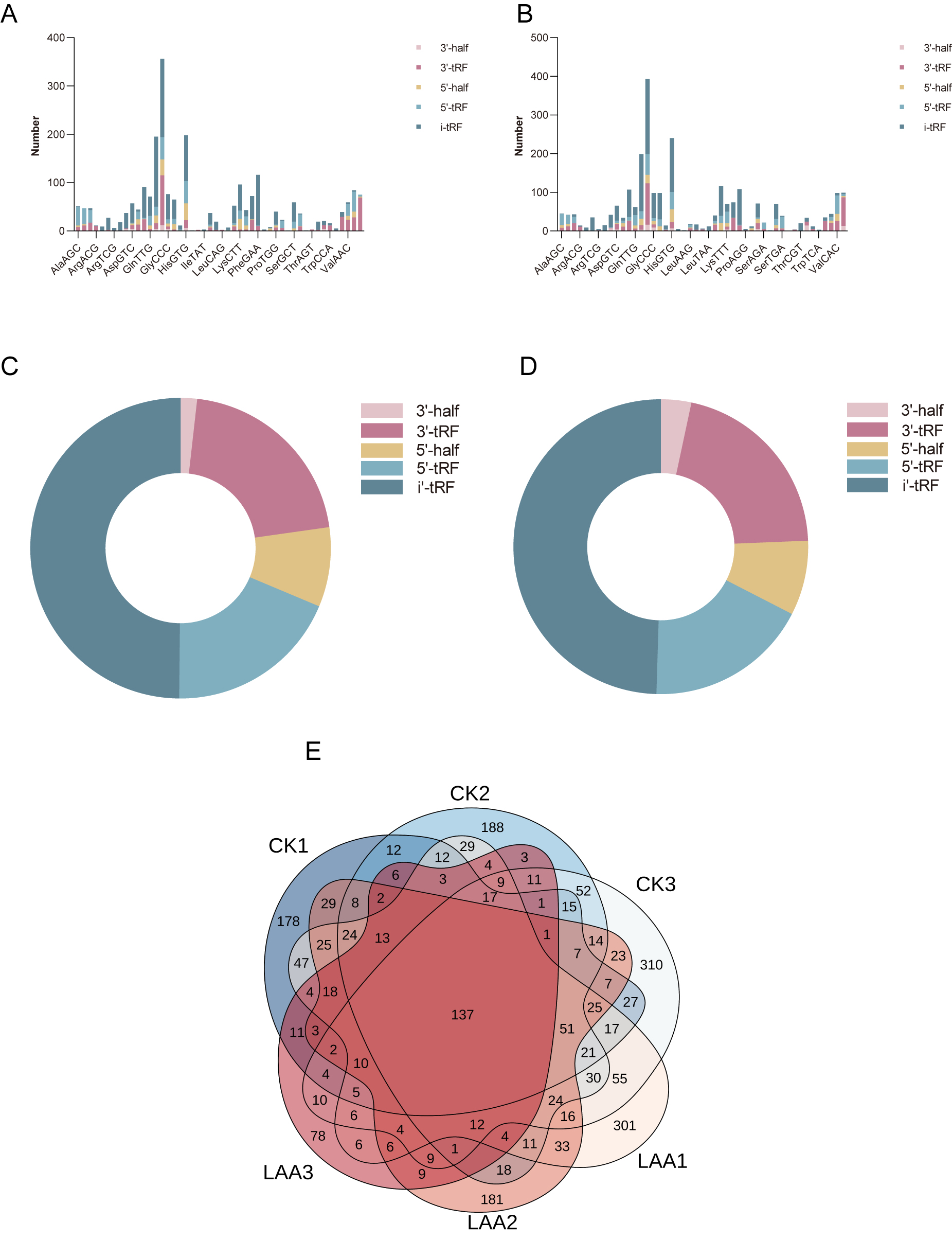
**

**FIGURE S1. RNA-seq results. (A)**. Subtypes and distribution of exosomes in LAA group **(B)** Subtypes and distribution of exosomes in NC group. **(C)** Pie chart of tsRNAs types in LAA group **(D)** Pie chart of tsRNAs types in NC group **(E)** Veen chart between LAA and CK(NC)





**Figure S2**. **Expression profiles of circulating exosome-tsRNAs. (A)** Heat map of tsRNAs in exosome RNA-seq. **(B)** Bar plot of the GO enrichment results (biological process, cellular component, and molecular function categories). **(C)** Bubble diagram for KEGG enrichment analysis.

### Supplementary Background

Stroke is the third most common disease in the world, and it’s high disability rates pose a high risk worldwide ^1^. In China, ischemic stroke (IS) occurrence increased the most (226.5%) among all types of strokes from 1990 to 2019 ^2^. According to the Trial of Org 10,172 in Acute Stroke Treatment (TOAST) classification, large-artery atherosclerotic (LAA) stroke forms the largest proportion of ischemic stroke (IS) ^3,4^, and rupture of atherosclerotic plaque is the main cause of LAA stroke ^5^. Advanced computed tomography (CT) and magnetic resonance imaging (MRI) have been widely applied to diagnose strokes ^6^; however, MRIs, which are more accurate, are not readily available in many centers ^7^. Moreover, traditional imaging has not been able to identify unstable plaques in the early stage. Therefore, it is essential to find a new approach that can provide more coverage, be more cost-effective, and predict the poor prognosis of LAA stroke and the risk of plaque rupture for early intervention. The more readily available humoral-related biomarkers have attracted extensive attention in recent years ^8,9^.

Recently, tsRNAs, previously thought to be a degradation product, have been implicated in many diseases, such as cancer, lupus nephritis, and coronary heart disease ^10-12^. tsRNAs (approximately 18–40 nt) is a kind of ancient small RNA produced by tRNA cleavage, which widely exists in various life forms ^13^. The functions of tsRNAs include gene silencing, ribosome biogenesis, retrotransposition, and epigenetic inheritance, which are rooted in its sequence conservation, RNA modification, and protein binding ability ^13,14^. Studies have shown that tsRNAs exhibit higher stability and expression levels than miRNA in mouse serum ^15,16^. The TRY-RNA (ts/rs/ysRNAs) signature is superior to miRNA-based biomarkers in differentiating pulmonary tuberculosis from lung cancer ^17^. However, the degradation of abundant biological enzymes in the serum and the restriction of tsRNAs source identification will inevitably impact the detection of tsRNAs. In contrast, the advantages of exosomal phospholipid bilayer protection and its ability to reflect many characteristics of the cells secreting them make exosomes promising in diagnosing and treating various diseases ^18^.

Exosomes are extracellular vesicles with a diameter of 60–140 nm that can be produced by various cells and carry nucleic acids, proteins, lipids, and metabolites. Exosome-mediated intercellular communication is reflected in various diseases, and we summarized the role of macrophage-related exosomes in the development of atherosclerosis (AS) ^18,19^. There are many small RNAs in exosomes, including miRNAs and tsRNAs ^18^. Compared with the more studied free RNA in serum, the RNA in exosomes shows more stable properties and traceability ^20^. The study of exosomal tsRNAs in esophageal cancer and HBV-related acute-on-chronic liver failure showed that exosomal tsRNAs have great potential as biomarkers for disease diagnosis ^21,22^. However, there are no studies on exosomal tsRNAs as biomarkers for stroke.

Our study indicated that targeting circulating exosomal tsRNAs is a potential strategy for LAA stroke diagnosis, short-term prognosis assessment, and plaque stability detection. In this study, exosomal tsRNAs differentially expressed between healthy people and LAA stroke patients were obtained by sequencing. Validation cohorts and repeat cohorts were validated, and subsequent diagnostic efficacy analyses were performed. We also compared the diagnostic performance of plasma and exosomal tsRNAs.

### Supplementary materials and methods

### Study population and clinical samples collection

Clinical samples for this study were collected in the Affiliated Hospital of Qingdao University, and a total of 506 samples were collected according to the protocol approved by the ethics committee from January 2019 to December 2020. After signing informed consent, peripheral blood samples were collected from 263 patients with AIS and the AS group (n=105, intracranial and extracranial arterial stenosis > 50% without symptoms) at the Department of Neurology, Affiliated Hospital of Qingdao University. The AIS group was divided into the LAA and SAO groups according to TOAST classification criteria ^4^. Subjects were recruited strictly according to the following inclusion criteria: (1) AIS diagnosed by craniocerebral CT or MRI (2) definite diagnosis within 3 days after the onset of symptoms. Individuals with severe heart disease, other systemic diseases, tumors, stroke with other TOAST subtypes, and cerebral embolism due to embolus detachment were excluded ^23,24^. Standard control volunteers were recruited from the Physical Examination Center of the Affiliated Hospital of Qingdao University. The inclusion criteria were healthy individuals with age, sex, body mass index, and history of smoking and drinking corresponding to the LAA stroke group ^24,25^. The blood samples were all fasting venous blood collected within 24 hours after admission, placed in a vacuum collection vessel containing EDTA, and centrifuged at 3000 rpm for 15 minutes at 4 ° C. The supernatant (plasma) was then collected into RNase-free EP tubes and stored at -80 ° C until analysis.

### Exosome isolation and purification

Exosomes were extracted and validated according to the protocol of the International Society for Extracellular Vesicles ^26^. Exosomes were isolated using an exosome extraction kit (Cat 4484450, Invitrogen Carlsbad, United States) ^27^. The process was as follows: the plasma was centrifuged at 2000×g and then at 10000×g for 20 min at ambient temperature to remove cells and debris. Then, 1 mL of centrifuged plasma was extracted, and 0.5 mL of phosphate-buffered saline (PBS) and 50 uL of proteinase K were added, thoroughly mixed, and incubated at 37 ° c for 10 min. After incubation, 0.3 mL exosome extraction reagent was added, mixed, and set at 4℃ for 30 minutes. Finally, after centrifugation at 10000×g for 5 min, the exosomes were re-suspended in PBS.

### Transmission Electron Microscopy for Exosome Identification

A total of 10 uL isolated exosome solution was placed on a copper net and incubated for 2 min at 25 ℃. Phosphotungstic acid (2%) was added for staining, followed by washing with sterile distilled water. Finally, the morphology of exosomes was observed using transmission electron microscopy (Hitachi, Tokyo, Japan) ^28^.

### Nanoparticle Tracking Analysis

Exosome particle size and number were analyzed using ZetaView PMX 110(Particle Metrix, Meerbusch, Germany) and Network Traffic Analysis (NTA) software (ZetaView 8.04.02) ^28^.

### Western Blot Analysis

Exosomes were lysed with RIPA and PMSF buffers to obtain total protein. After gel electrophoresis (10% SDS-PAGE) and membrane transfer, the total protein was transferred to the PVDF membrane and incubated overnight at 4 ° C with anti-exosome specific markers (positive: CD9, CD63, and TSG101; Negative: GRP94) primary antibodies (Ab92726, Ab134045, Ab125011, and Ab238126; Abcam, Cambridge, UK) and then incubated with goat anti-rabbit secondary antibody (Abcam, Cambridge, UK) for 1 hour before detection of exosomal protein expression ^26,28,29^.

### Small RNA Library Construction, Sequencing, and Data Analysis

### We extracted and purified total RNA from the exosomes of the discovery cohort (LAA=3, NC=3) using the miRNasy Serum/Plasma Advanced Kit. Highly sensitive Agilent 2100 PIC600 was used to detect the total amount and fragment distribution of RNA accurately. After the Sample was qualified, the Small RNA Sample Pre Kit was used to construct the library. Clean data were obtained after filtering the raw data obtained by sequencing. The clean reads of each sample were screened for sRNA within a specific length range (16–50 nt) for tsRNAs prediction. tsRNAs were identified using Mintmap software. The tsRNAs that only come from the tRNA region are called exclusive-tRFs, and the tsRNAs that are compared to other regions of the genome besides the tRNA region are called ambiguity-tRFs. The exclusive-tRFs (tsRNAs) were selected for downstream analysis. TMM was used to standardize ReadCount data, and DEGseq was used for differential analysis. tsRNAs with qvalue< 0.01 and |fold change| ≥2.0 (log2 ratio ≥1.0 or ≤ - 1.0) were regarded as differentially expressed, and the result was represented by a volcano diagram.

### Gene Ontology (GO) and Kyoto Encyclopedia of Genes and Genomes (KEGG) Functional Enrichment Analysis

The intersection of miRanda and RNAhybrid targeting software was used as the target gene of tsRNAs for downstream analysis ^11,30^. According to the correspondence between tsRNAs and its target genes, we performed GO and KEGG enrichment analysis for each set of target genes of differentially expressed tsRNAs ^31^. GOseq R package was used for GO enrichment analysis. KEGG pathway enrichment was analyzed using KEGG Orthology Based Annotation System.

**Quantification of tsRNA Expression With RT-qPCR**

Total RNA was extracted and purified from plasma exosomes according to the manufacturer's protocol ^32^. Reverse transcription of tsRNAs was performed to obtain cDNA using the Mir-X miRNA First-strand Synthesis Kit (Takara, Japan). TB-Green PreMix Ex Taq™II (Takara, Japan) was used for quantitative amplification. The expression level of the reference gene U6 was used to normalize the relative expression level of tsRNAs, and fold changes of tsRNAs expression were calculated using the following formula ^21^:

2 ^-ΔΔCt^

Primer sequences are described in the Supplementary Material (**Table S1**).

### Statistical Analysis

Categorical variables in the clinical baseline table are expressed as percentages. For continuous variables, normally distributed variables are expressed as mean ±SEM; Variables with skewed distributions are expressed as quartiles. Continuous variables were calculated by analysis of variance, t-, Mann–Whitney U, and Kruskal–Wallis tests. The Chi-square test was used to calculate categorical variables. Correlation analysis was performed using Spearman test. Univariate and multivariate binary logistic regression analyses were used to establish the regression model. ROC curve, Net Reclassification Improvement (NRI), and Integrated Discriminant Improvement (IDI) were used to evaluate the diagnostic efficiency of the prediction model. All data were analyzed using the following software: SPSS 26.0, GraphPad Prism 9.0, and R version 4.2.1. For all analyses, P＜0.05 was considered statistically significant. VennDiagram, pheatmap, and ggplot2 R packages were used for the visualization of results.

### Supplementary discussion

In this study, we obtained circulating exosomal tsRNAs expression profiles of LAA patients and healthy control volunteers by RNA-seq. Combined with foldchange and functional enrichment results, we screened five exosomes for a cohort study. We found that exosomal tsRNAs can be used as a biomarker for LAA stroke diagnosis and short-term prognosis and for predicting the risk of plaque rupture.

Studies on tsRNAs as disease biomarkers have focused on directly determining plasma tsRNAs expression profiles ^33-35^. Plasma tsRNAs can originate from various cells, but this free form also makes tracing its origin difficult. In contrast, the phospholipid bilayers of exosomes have many protein markers that reflect their source cells ^18^. The vesicle structure also isolates tsRNAs from biological enzymes in plasma, making it more stable ^18^. Therefore, we selected direct sequencing of circulating exosomal tsRNAs to obtain the difference in tsRNAs expression levels between LAA stroke and normal control groups.

Further analysis of the obtained significantly different exosomal tsRNAs revealed that the target genes of tRF-19-INVDRIFU were associated with inflammation, such as MAPK9 and STAT1 ^36-38^. The inflammatory mechanism is closely related to AS ^39^. Combined with fold change and enrichment analysis results, we screened five tsRNAs related to the pathological process of LAA stroke, which were tRF-19-INVDRIFU, tRF-31-FN8DYDZDL9X1B, tRF-34-79MP9PMNH5IS15, and tRF-31-FN8DYDZDL9X1B， tRF - 36 - FN7BWU2F5JYH0RE and tRF - 38 - Q99P9P9NH57S36D1. Among them, the target gene of tRF-31-FN8DYDZDL9X1B is WNT5A, a non-classical WNT, and Loannis et al. summarized its role in cardiovascular diseases in detail ^40^. In addition, it has been reported that silencing NTN1 can induce inflammation and plaque regression ^41^, and in our analysis, NTN1 is the target gene of tRF-38-Q99P9P9NH57S36D1. Therefore, we selected the above candidate tsRNAs for further analysis based on the prediction of target gene function.

In the differential tsRNAs validation phase based on two cohorts (a small cohort in the validation phase and a large cohort in the repeat phase), we included the SAO group to exclude interference from the differential profile of AIS ^24,42^. RT-qPCR analysis showed that tRF-19-INVDRIFU, tRF-31-FN8DYDZDL9X1B, tRF-34-79MP9PMNH5IS15, and tRF-38-Q99P9P9NH57S36D1 could simultaneously distinguish LAA stroke from healthy control and SAO stroke groups. To further evaluate the diagnostic efficacy of exosomal tsRNAs, we constructed a diagnostic model, and the results showed that exo-tRF-19-INVDRIFU was an independent risk factor for LAA stroke, and tRF-38-Q99P9P9NH57S36D1 had a protective effect, both of which had good diagnostic efficacy. Moreover, the combined diagnostic model was superior to single exosomal tsRNAs.

Previous studies have often focused on plasma-derived biomarkers ^25,43^. By comparing the results of ROC, we aimed to determine that circulating exosomal tsRNAs are superior to plasma tsRNAs in diagnosing LAA stroke. The results showed that only plasma tRF-31-FN8DYDZDL9X1B and plasma tRF-38-Q99P9P9NH57S36D1 could distinguish the LAA group from the NC group and SAO group at the same time, and the AUC of the diagnostic model constructed by them was lower than that of the corresponding exosomal tsRNAs. These results suggest that circulating exosomal tsRNAs have more potential as a biomarker for LAA stroke.

Subsequently, we also analyzed the correlation between NIHSS score and exosomal tsRNAs expression level. We found that the expression level of exo-tRF-19-INVDRIFU was positively correlated with NIHSS, while that of exo-tRF-38-Q99P9P9NH57S36D1 was negatively correlated. These results suggest that exosomal tsRNAs play a role in assessing stroke severity. The assessment of prognosis is a reference for the choice of treatment. mRS Score is a widely used quantitative standard for prognosis assessment in clinics ^44^. We further analyzed the relationship between mRS Score (1 month after discharge) and exosomal tsRNAs expression levels to determine whether exosomal tsRNAs can be used as biomarkers to assess short-term prognosis. The results showed that the expression levels of exosomes tRF-19-INVDRIFU and tRF-38-Q99P9P9NH57S36D were significantly different between the poor and good prognosis groups. These results suggest that exo-tRF-19-INVDRIFU and exo-tRF-38-Q99P9P9NH57S36D might be novel biomarkers for evaluating short-term prognosis.

The rupture of unstable plaques is the leading cause of LAA stroke ^45^. Studies on identifying unstable plaques mainly evaluated plaque stability by defining an index ^46,47^. Recently, it has also been shown that CD68, PAM, and IGFBP6 can be used as diagnostic markers to identify unstable plaques ^48^. To determine whether exosomal tsRNAs could be novel biomarkers for predicting unstable plaques, we included the AS group in duplicate cohorts. Interestingly, exo-tRF-19-INVDRIFU was an independent risk factor for plaque rupture. It also did a good performance in assessing LAA stroke severity and short-term outcome. Our findings may provide a method for early warning of LAA stroke.

The limitation of our experiment naturally lies in the sample source and sample size. The selection may bias a sample from a single region, hence a multicenter study with a larger sample is relatively more convincing. However, our study also provides data support for future multicenter studies. In the future, we will continue to study the mechanism of exosomal tsRNAs in the pathological process of LAA to reveal the mystery between exosomal tsRNAs and LAA stroke.

In general, circulating exosomal tsRNAs were superior to plasma tsRNAs as novel biomarkers for LAA stroke diagnosis. Furthermore, it was also found to have great potential to assess the severity, short-term prognosis, and plaque stability of LAA stroke. Thus, it provides a new perspective for the diagnosis and prevention of LAA stroke.

### Abbreviations

AIS, Acute ischemic stroke; tsRNAs, tRNA-derived small RNAs; LAA, large-artery atherosclerotic; AS, atherosclerosis; SAO, small artery occlusion; TOAST, Trial of Org 10,172 in Acute Stroke Treatment; IS, ischemic stroke; CT, computed tomography; MRI, magnetic resonance imaging; HBV, hepatitis B virus; EDTA, Ethylene Diamine Tetraacetic Acid; GO, Gene Ontology; KEGG, Kyoto Encyclopedia of Genes and Genomes; NTA, Nanoparticle Tracking Analysis; RT-qPCR, real-time quantitative polymerase chain reaction; ROC, receiver operating characteristic curves; NRI, Net Reclassification Improvement; IDI, Integrated Discriminant Improvement; LDL, low-density lipoprotein; TG, triglycerides; TC, total cholesterol; LDL, low-density lipoprotein; NIHSS, National Institutes of Health Stroke Score Scale; AUC, areas under the curves; mRS, modified Rankin Scale;

### Author contributions

All authors read and approved the final version of the manuscript. XYZ, XDP, RYH were involved in the study design. KYY, QX, KW, JZ collected the samples, performed the experiments and analyzed the data. KYY, QX completed the manuscript. All authors contributed to the article and approved the submitted version.

### Conflict of interests

Authors declare no conflict of interests.

### Acknowledgment

We thank all members of the Department of Neurology at The Affiliated Hospital of Qingdao University, especially all nurses, for assistance with blood withdrawal.

### Funding

This work was supported by National Natural Science Foundation of China (No.82171299).

### Data Availability

The data that support the findings of this study are available from the corresponding author upon reasonable request.

### References

1. Global burden of 369 diseases and injuries in 204 countries and territories, 1990-2019: a systematic analysis for the Global Burden of Disease Study 2019. *Lancet (London, England)*. Oct 17 2020;396(10258):1204-1222. doi:10.1016/s0140-6736(20)30925-9

2. Ma Q, Li R, Wang L, et al. Temporal trend and attributable risk factors of stroke burden in China, 1990-2019: an analysis for the Global Burden of Disease Study 2019. *The Lancet Public health*. Dec 2021;6(12):e897-e906. doi:10.1016/s2468-2667(21)00228-0

3. Ornello R, Degan D, Tiseo C, et al. Distribution and Temporal Trends From 1993 to 2015 of Ischemic Stroke Subtypes: A Systematic Review and Meta-Analysis. *Stroke*. Apr 2018;49(4):814-819. doi:10.1161/strokeaha.117.020031

4. Adams HP, Jr., Bendixen BH, Kappelle LJ, et al. Classification of subtype of acute ischemic stroke. Definitions for use in a multicenter clinical trial. TOAST. Trial of Org 10172 in Acute Stroke Treatment. *Stroke*. Jan 1993;24(1):35-41. doi:10.1161/01.str.24.1.35

5. Libby P. The changing landscape of atherosclerosis. *Nature*. Apr 2021;592(7855):524-533. doi:10.1038/s41586-021-03392-8

6. Lin MP, Liebeskind DS. Imaging of Ischemic Stroke. *Continuum (Minneapolis, Minn)*. Oct 2016;22(5, Neuroimaging):1399-1423. doi:10.1212/con.0000000000000376

7. Fisher M, Saver JL. Future directions of acute ischaemic stroke therapy. *The Lancet Neurology*. Jul 2015;14(7):758-67. doi:10.1016/s1474-4422(15)00054-x

8. Li W, Qi Z, Kang H, et al. Serum Occludin as a Biomarker to Predict the Severity of Acute Ischemic Stroke, Hemorrhagic Transformation, and Patient Prognosis. *Aging and disease*. Dec 2020;11(6):1395-1406. doi:10.14336/ad.2020.0119

9. Liu Y, Li Y, Zang J, et al. CircOGDH Is a Penumbra Biomarker and Therapeutic Target in Acute Ischemic Stroke. *Circulation research*. Mar 18 2022;130(6):907-924. doi:10.1161/circresaha.121.319412

10. Wang Y, Weng Q, Ge J, Zhang X, Guo J, Ye G. tRNA-derived small RNAs: Mechanisms and potential roles in cancers. *Genes & diseases*. Nov 2022;9(6):1431-1442. doi:10.1016/j.gendis.2021.12.009

11. Yang P, Zhang X, Chen S, et al. A Novel Serum tsRNA for Diagnosis and Prediction of Nephritis in SLE. *Frontiers in immunology*. 2021;12:735105. doi:10.3389/fimmu.2021.735105

12. Wang S, Luo Z, Yuan L, et al. tRNA-Derived Small RNAs: Novel Insights into the Pathogenesis and Treatment of Cardiovascular Diseases. *Journal of cardiovascular translational research*. Oct 3 2022;doi:10.1007/s12265-022-10322-0

13. Chen Q, Zhang X, Shi J, Yan M, Zhou T. Origins and evolving functionalities of tRNA-derived small RNAs. *Trends in biochemical sciences*. Oct 2021;46(10):790-804. doi:10.1016/j.tibs.2021.05.001

14. Liu B, Cao J, Wang X, Guo C, Liu Y, Wang T. Deciphering the tRNA-derived small RNAs: origin, development, and future. *Cell death & disease*. Dec 21 2021;13(1):24. doi:10.1038/s41419-021-04472-3

15. Zhang Y, Zhang Y, Shi J, et al. Identification and characterization of an ancient class of small RNAs enriched in serum associating with active infection. *Journal of molecular cell biology*. Apr 2014;6(2):172-4. doi:10.1093/jmcb/mjt052

16. Victoria B, Dhahbi JM, Nunez Lopez YO, et al. Circulating microRNA signature of genotype-by-age interactions in the long-lived Ames dwarf mouse. *Aging cell*. Dec 2015;14(6):1055-66. doi:10.1111/acel.12373

17. Gu W, Shi J, Liu H, et al. Peripheral blood non-canonical small non-coding RNAs as novel biomarkers in lung cancer. *Molecular cancer*. Nov 12 2020;19(1):159. doi:10.1186/s12943-020-01280-9

18. Kalluri R, LeBleu VS. The biology, function, and biomedical applications of exosomes. *Science (New York, NY)*. Feb 7 2020;367(6478)doi:10.1126/science.aau6977

19. Yang K, Xiao Q, Niu M, Pan X, Zhu X. Exosomes in atherosclerosis: Convergence on macrophages. *International journal of biological sciences*. 2022;18(8):3266-3281. doi:10.7150/ijbs.71862

20. Colao IL, Corteling R, Bracewell D, Wall I. Manufacturing Exosomes: A Promising Therapeutic Platform. *Trends in molecular medicine*. Mar 2018;24(3):242-256. doi:10.1016/j.molmed.2018.01.006

21. Li K, Lin Y, Luo Y, et al. A signature of saliva-derived exosomal small RNAs as predicting biomarker for esophageal carcinoma: a multicenter prospective study. *Molecular cancer*. Jan 18 2022;21(1):21. doi:10.1186/s12943-022-01499-8

22. Xu W, Yu M, Wu Y, et al. Plasma-Derived Exosomal SncRNA as a Promising Diagnostic Biomarker for Early Detection of HBV-Related Acute-on-Chronic Liver Failure. *Frontiers in cellular and infection microbiology*. 2022;12:923300. doi:10.3389/fcimb.2022.923300

23. Shen Y, Peng C, Bai Q, et al. Epigenome-Wide Association Study Indicates Hypomethylation of MTRNR2L8 in Large-Artery Atherosclerosis Stroke. *Stroke*. Jun 2019;50(6):1330-1338. doi:10.1161/strokeaha.118.023436

24. Xiao Q, Hou R, Li H, et al. Circulating Exosomal circRNAs Contribute to Potential Diagnostic Value of Large Artery Atherosclerotic Stroke. *Frontiers in immunology*. 2021;12:830018. doi:10.3389/fimmu.2021.830018

25. Zuo L, Zhang L, Zu J, et al. Circulating Circular RNAs as Biomarkers for the Diagnosis and Prediction of Outcomes in Acute Ischemic Stroke. *Stroke*. Jan 2020;51(1):319-323. doi:10.1161/strokeaha.119.027348

26. Théry C, Witwer KW, Aikawa E, et al. Minimal information for studies of extracellular vesicles 2018 (MISEV2018): a position statement of the International Society for Extracellular Vesicles and update of the MISEV2014 guidelines. *Journal of extracellular vesicles*. 2018;7(1):1535750. doi:10.1080/20013078.2018.1535750

27. Tian Y, Gong M, Hu Y, et al. Quality and efficiency assessment of six extracellular vesicle isolation methods by nano-flow cytometry. *Journal of extracellular vesicles*. 2020;9(1):1697028. doi:10.1080/20013078.2019.1697028

28. Min L, Zhu S, Chen L, et al. Evaluation of circulating small extracellular vesicles derived miRNAs as biomarkers of early colon cancer: a comparison with plasma total miRNAs. *Journal of extracellular vesicles*. 2019;8(1):1643670. doi:10.1080/20013078.2019.1643670

29. Saenz-Pipaon G, San Martín P, Planell N, et al. Functional and transcriptomic analysis of extracellular vesicles identifies calprotectin as a new prognostic marker in peripheral arterial disease (PAD). *Journal of extracellular vesicles*. 2020;9(1):1729646. doi:10.1080/20013078.2020.1729646

30. Niu M, Li H, Li X, et al. Circulating Exosomal miRNAs as Novel Biomarkers Perform Superior Diagnostic Efficiency Compared With Plasma miRNAs for Large-Artery Atherosclerosis Stroke. *Frontiers in pharmacology*. 2021;12:791644. doi:10.3389/fphar.2021.791644

31. Mao X, Cai T, Olyarchuk JG, Wei L. Automated genome annotation and pathway identification using the KEGG Orthology (KO) as a controlled vocabulary. *Bioinformatics (Oxford, England)*. Oct 1 2005;21(19):3787-93. doi:10.1093/bioinformatics/bti430

32. Androvic P, Romanyuk N, Urdzikova-Machova L, Rohlova E, Kubista M, Valihrach L. Two-tailed RT-qPCR panel for quality control of circulating microRNA studies. *Scientific reports*. Mar 12 2019;9(1):4255. doi:10.1038/s41598-019-40513-w

33. Zhang Y, Gu X, Qin X, Huang Y, Ju S. Evaluation of serum tRF-23-Q99P9P9NDD as a potential biomarker for the clinical diagnosis of gastric cancer. *Molecular medicine (Cambridge, Mass)*. Jun 11 2022;28(1):63. doi:10.1186/s10020-022-00491-8

34. Xue M, Shi M, Xie J, et al. Serum tRNA-derived small RNAs as potential novel diagnostic biomarkers for pancreatic ductal adenocarcinoma. *American journal of cancer research*. 2021;11(3):837-848.

35. Zhang X, Yang P, Khan A, et al. Serum tsRNA as a novel molecular diagnostic biomarker for lupus nephritis. *Clinical and translational medicine*. May 2022;12(5):e830. doi:10.1002/ctm2.830

36. Hueso M, Mallén A, Casas Á, et al. Integrated miRNA/mRNA Counter-Expression Analysis Highlights Oxidative Stress-Related Genes CCR7 and FOXO1 as Blood Markers of Coronary Arterial Disease. *International journal of molecular sciences*. Mar 12 2020;21(6)doi:10.3390/ijms21061943

37. Fu X, Sun Z, Long Q, et al. Glycosides from Buyang Huanwu Decoction inhibit atherosclerotic inflammation via JAK/STAT signaling pathway. *Phytomedicine : international journal of phytotherapy and phytopharmacology*. Oct 2022;105:154385. doi:10.1016/j.phymed.2022.154385

38. Zhang M, Zhu Y, Zhu J, et al. circ_0086296 induced atherosclerotic lesions via the IFIT1/STAT1 feedback loop by sponging miR-576-3p. *Cellular & molecular biology letters*. Sep 23 2022;27(1):80. doi:10.1186/s11658-022-00372-2

39. Libby P. Inflammation during the life cycle of the atherosclerotic plaque. *Cardiovascular research*. Nov 22 2021;117(13):2525-2536. doi:10.1093/cvr/cvab303

40. Akoumianakis I, Polkinghorne M, Antoniades C. Non-canonical WNT signalling in cardiovascular disease: mechanisms and therapeutic implications. *Nature reviews Cardiology*. Jun 13 2022:1-15. doi:10.1038/s41569-022-00718-5

41. Schlegel M, Sharma M, Brown EJ, et al. Silencing Myeloid Netrin-1 Induces Inflammation Resolution and Plaque Regression. *Circulation research*. Aug 20 2021;129(5):530-546. doi:10.1161/circresaha.121.319313

42. Zhang S, Wang X, Yin R, et al. Circulating exosomal lncRNAs as predictors of risk and unfavorable prognosis for large artery atherosclerotic stroke. *Clinical and translational medicine*. Dec 2021;11(12):e555. doi:10.1002/ctm2.555

43. Zu J, Zuo L, Zhang L, et al. Circular RNA FUNDC1 for Prediction of Acute Phase Outcome and Long-Term Survival of Acute Ischemic Stroke. *Frontiers in neurology*. 2022;13:846198. doi:10.3389/fneur.2022.846198

44. Gumbinger C, Ringleb P, Ippen F, et al. Outcomes of patients with stroke treated with thrombolysis according to prestroke Rankin Scale scores. *Neurology*. Nov 12 2019;93(20):e1834-e1843. doi:10.1212/wnl.0000000000008468

45. Shaw LJ, Blankstein R, Min JK. Outcomes in Stable Coronary Disease: Is Defining High-Risk Atherosclerotic Plaque Important? *Journal of the American College of Cardiology*. Jan 29 2019;73(3):302-304. doi:10.1016/j.jacc.2018.11.017

46. Khosravi A, Sadeghi M, Farsani ES, et al. Atherogenic index of plasma: A valuable novel index to distinguish patients with unstable atherogenic plaques. *Journal of research in medical sciences : the official journal of Isfahan University of Medical Sciences*. 2022;27:45. doi:10.4103/jrms.jrms_590_21

47. Sagris M, Antonopoulos AS, Simantiris S, et al. Pericoronary fat attenuation index-a new imaging biomarker and its diagnostic and prognostic utility: a systematic review and meta-analysis. *European heart journal Cardiovascular Imaging*. Sep 7 2022;doi:10.1093/ehjci/jeac174

48. Wang J, Kang Z, Liu Y, Li Z, Liu Y, Liu J. Identification of immune cell infiltration and diagnostic biomarkers in unstable atherosclerotic plaques by integrated bioinformatics analysis and machine learning. *Frontiers in immunology*. 2022;13:956078. doi:10.3389/fimmu.2022.956078
